# Phage*Dive*: the comprehensive strain database of prokaryotic viral diversity

**DOI:** 10.1093/nar/gkae878

**Published:** 2024-10-07

**Authors:** Clara Rolland, Johannes Wittmann, Lorenz C Reimer, Joaquim Sardà Carbasse, Isabel Schober, Christian-Alexander Dudek, Christian Ebeling, Julia Koblitz, Boyke Bunk, Jörg Overmann

**Affiliations:** Leibniz Institute DSMZ-German Collection of Microorganisms and Cell Cultures GmbH, 38124 Braunschweig, Germany; Leibniz Institute DSMZ-German Collection of Microorganisms and Cell Cultures GmbH, 38124 Braunschweig, Germany; Leibniz Institute DSMZ-German Collection of Microorganisms and Cell Cultures GmbH, 38124 Braunschweig, Germany; Leibniz Institute DSMZ-German Collection of Microorganisms and Cell Cultures GmbH, 38124 Braunschweig, Germany; Leibniz Institute DSMZ-German Collection of Microorganisms and Cell Cultures GmbH, 38124 Braunschweig, Germany; Leibniz Institute DSMZ-German Collection of Microorganisms and Cell Cultures GmbH, 38124 Braunschweig, Germany; Leibniz Institute DSMZ-German Collection of Microorganisms and Cell Cultures GmbH, 38124 Braunschweig, Germany; Leibniz Institute DSMZ-German Collection of Microorganisms and Cell Cultures GmbH, 38124 Braunschweig, Germany; Leibniz Institute DSMZ-German Collection of Microorganisms and Cell Cultures GmbH, 38124 Braunschweig, Germany; Leibniz Institute DSMZ-German Collection of Microorganisms and Cell Cultures GmbH, 38124 Braunschweig, Germany

## Abstract

Prokaryotic viruses represent the most diverse and abundant biological entities on Earth. So far, data on bacteriophages are not standardized, not readily available for comparative analyses and cannot be linked to the rapidly growing (meta)genomic data. We developed Phage*Dive* (https://phagedive.dsmz.de), a comprehensive database for prokaryotic viruses gathering all existing data dispersed across multiple sources, like scientific publications, specialized databases or internal files of culture collections. Phage*Dive* allows to link own research data to the existing information through an easy and central access, providing fields for various experimental data (host range, genomic data, etc.) and available metadata (e.g. geographical origin, isolation source). An important feature is the link between experimental data, the culture collection number and the repository of the corresponding physical bioresource. To date, Phage*Dive* covers 1167 phages from three different world-renowned public collections (DSMZ, Félix d’Hérelle Reference Center for Bacterial Viruses and NCTC) and features an advanced search function using all data fields from the sections like taxonomy or morphology by controlled vocabulary and ontologies. Phage*Dive* is fully interoperable with other resources including NCBI, the Viral Host Range database (VHRdb) of Institute Pasteur or the Bac*Dive* and Media*Dive* databases of DSMZ.

## Graphical Abstract

**Graphical Abstract:**
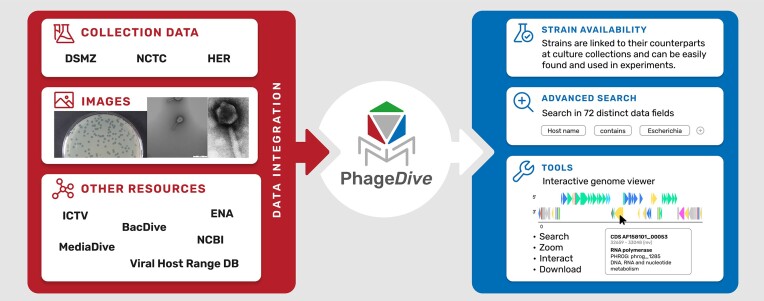

## Introduction

Since their discovery by Félix d′Hérelle and Fredrick Twort over a century ago >11 200 bacteriophage species have been characterized (1) which makes them the largest virus group known to date. The vast majority of the known bacteriophages are tailed and contain double-stranded DNA (2,3). Culture-independent molecular approaches have revealed that bacteriophages occur in a much larger diversity and in high abundance in essentially all environments on Earth, ranging from marine waters (4) and soils (5), to acidic hot volcanic springs (6) and the human gut (7). The crude estimates of total viral diversity amount to 10^7^–10^9^ virus species (8). At present, only a very limited fraction of this existing bacteriophage diversity is maintained in public collections worldwide, which provide the quality-controlled and documented cultures needed for scientific studies of bacteriophage biology. Besides the isolation, characterization, biobanking, and genome analysis of bacteriophages, an easy access to structured (meta)data associated with the different phage species is essential for future studies, particularly for comparative analyses.

The current release on the update of virus taxonomy of the International Committee on Taxonomy of Viruses (ICTV) comprises 11 273 virus species in 2818 genera and 264 families (1). For these viruses, the information on the names, taxonomy, morphology, genome features, proteins, host range and origin can be found in the report chapters of the ICTV database (report on Virus Classification and Taxon Nomenclature, https://ictv.global/taxonomy/history, Retrieved 19 February 2024). However, this open access, public database is organized along taxonomic levels and does not use controlled vocabulary or ontologies. Most of the existing databases on bacteriophages focus on collecting and providing genomic data like genetic features or predicted lifestyles (e.g. PhageScope (9)), or on specific aspects of phage biology like virus-host interactions like MVP or VHRdb (10,11). The database PhagesDB collects and shares data exclusively for actinobacterial phages (12). The European Virus Archive Global (https://www.european-virus-archive.com/evag-portal) only covers human and animal pathogens. Hence, no database exists so far which can accommodate additional metadata on bacteriophages from literature or would enable specific searches for single features (e.g. a search term ‘phage’ to extract viruses that infect bacteria or archaea) or for specific combinations of characteristics (e.g. ‘host + marine + *Roseobacter*’, to recover all bacteriophages infecting bacteria of the genus *Roseobacter*; or ‘DNA + host = *Staphylococcus*’, to recover all DNA viruses infecting *Staphylococcus*).

We established the database Phage*Dive* (https://phagedive.dsmz.de) that provides comprehensive information on bacteriophage biology, molecular mechanisms of host interaction, or phage ecology and links this information to established bacteriophage taxa.

## Materials and methods

### Technical information

The Graphical User Interface (GUI) of Phage*Dive* was individually built with PHP 8.2. The frontend relies on JavaScript and JQuery. For better usability, the GUI only displays filled data fields and hides empty ones. Therefore, the Phage*Dive* GUI might appear differently from strain to strain. The relational database engine is MariaDB 10.8.3. The database scheme consists of 156 data fields stored in 17 tables. This technical framework ensures the flexibility to keep up with the growing database and allows to integrate new data types and to adapt the GUI according to user feedback and needs.

## Results

### User interface

Currently, Phage*Dive* harbours data for bacteriophages and archaeal viruses which are physically available in public culture collections such as the Félix d’Hérelle Reference Center for Bacterial Viruses in Canada, the National Collection of Type Cultures (NCTC) in UK and DSMZ phage collections (Germany) amounting to a total of 1167 viruses. The decision to base Phage*Dive* on culture collections was taken because the access to the physical resources of viruses and their hosts enables future analysis, thereby ensuring reproducibility and improvement of the quality of the research data. All information collected through the culture collections and other various sources such as genomic resources or literature was standardized and classified into 99 data fields, grouped into 12 categories.

### Individual strain page content

Phage*Dive* offers entries for each individual virus strain, aggregating the available data and directly linking them to strain identifiers like official collection numbers of the original source, and catalogue entries of the respective collection, if available.

**Figure 1.**
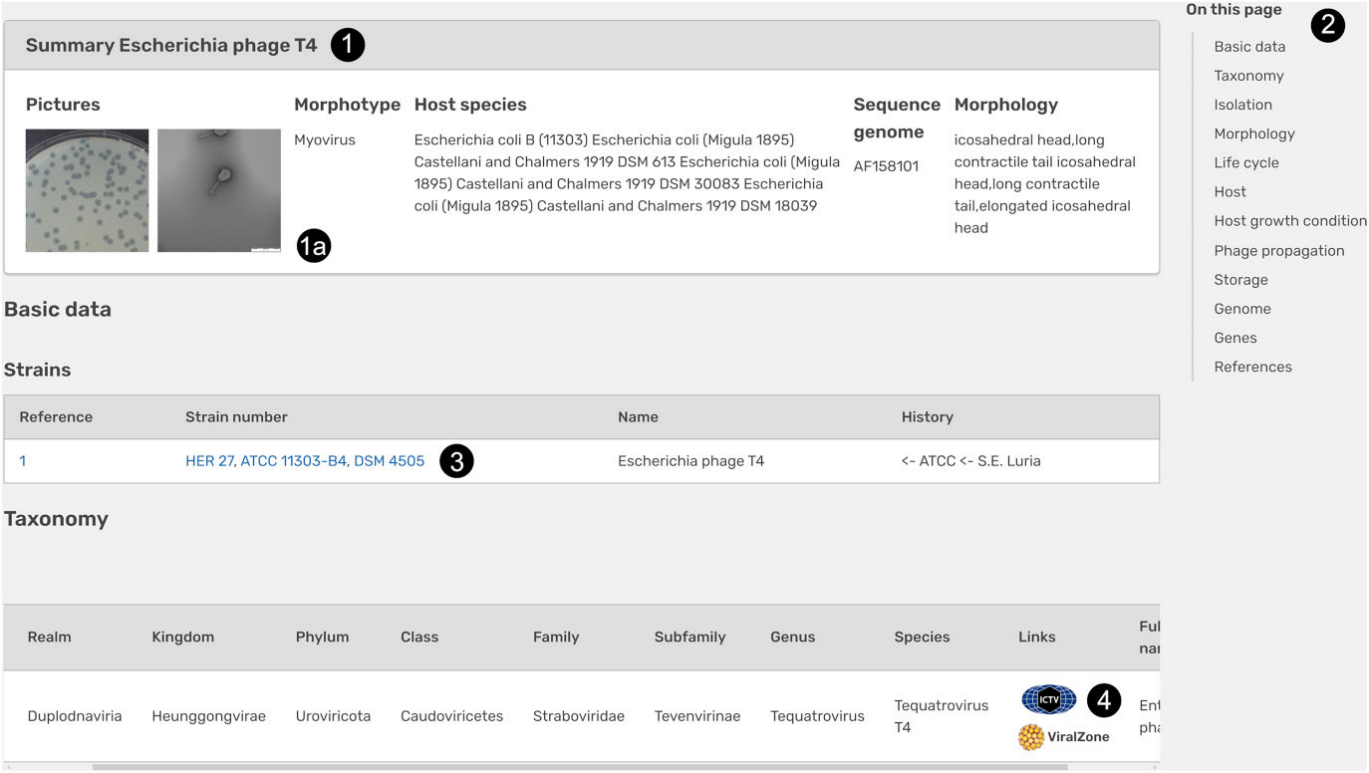
Example of an individual strain page (*Escherichia* phage T4, https://phagedive.dsmz.de/strain/160).

The individual pages for the viruses are structured by section as follows (Figure 1). A summary provides key data like name, morphotype, host species, as well as data on the morphology and pictures of phage plaques on a host culture, and electron photomicrographs, if available (Figure 1, section 1, label 1a). All sections displayed for a virus are listed in the right-hand upper corner (Figure 1, label 2). Basic data include strain identifiers directly linked to respective catalogues, the name of the strain and strain history (Figure 1, label 3). Data included in Phage*Dive* cover basic information such as geographic origin, isolation sources, morphology, lifestyle, taxonomy (Figure 1, label 4), and associated literature. Particular attention was paid to virus morphology. Since current taxonomy focuses mainly on the genomic and proteomic data and phylogenetic relationships of viruses and in this course families reflecting morphological features of their members directly in their names like *Myoviridae* or *Siphoviridae* were recently abolished (13), this previous classification based on morphology is depicted in the morphotype section (e.g. siphovirus, myovirus, podovirus etc.) to maintain consistency of Phage*Dive* with previous data. These morphotypes and the morphological description of plaques are supported by the transmission electron micrographs (TEM) and plaques pictures (Figure 1, label 1a). The latter can be enlarged interactively, providing good clarity. For detailed information on virus propagation, we also provide data on the recommended host strains and cultivation media through direct links to Bac*Dive* (14) and Media*Dive* (15), respectively. Information on virus-host interactions is based on internal host range data of the DSMZ as well as on the Viral Host Range Database hosted by the Pasteur Institute in Paris (11) . In addition, general genomic information like accession number, genome length or GC content are directly displayed on the virus-specific webpage. If available, genome sequences with GenBank accessions were retrieved from NCBI, reannotated using Prokka version 1.14.6 (16) and the PHROG database (17) and implemented into a genome viewer tool.

As a strain page may aggregate data from different sources, each data entry of the Phage*Dive* database is labeled by a reference number to the left (Figure 1, label 3;). This number is linked to the reference section at the bottom of the virus description page. Through this extensive documentation users can easily retrace the origin of the data.

For a better usability of the interface, only section and data fields for which data is available are presented.

### Linking and integrating available data sources

One of the main characteristics of Phage*Dive* is to integrate and standardize the highly dispersed data on bacteriophages and to link them to the bioresource of origin. Depending on the source, data are either directly integrated into Phage*Dive* and provided in a standardized format, or data are made accessible via direct links to the external sources to provide the user with the access to associated information. The former typically is the case for internal data from culture collections that are integrated in standardized data fields, which enables data comparison and systematic searches. For transparency, every single data point is associated with a reference id linking to the original source. Genome sequence data are reannotated and provided with the genome viewer. Other data types, in particular sequence data, bacterial strain data or cultivation media data, are in a highly specific, stable format and readily available through well-established, active databases. In these cases, data are directly linked to the respective web resource, allowing users to easily explore these data while ensuring access to the regularly updated information provided by the external sources. Important external sources directly linked in Phage*Dive* are NCBI for sequence data, Bac*Dive* for bacterial strain data, Media*Dive* for cultivation media recipes, Viral Host Range database for virus host range data, ICTV for taxonomy, ViPTree for an overview on the phage phylogeny (18) and ViralZone (19) for general properties of taxonomic groups like genome features, further information on replication or putative receptors.

In turn, Phage*Dive* strains are indexed and linked by other web resources which increases visibility and findability. As a part of the newly established, overarching biodata infrastructure DSMZ Digital Diversity, viruses are linked to their respective host strain in Bac*Dive* and also indexed in the federated search of the DSMZ Digital Diversity Hub page.

### Tools

**Figure 2.**
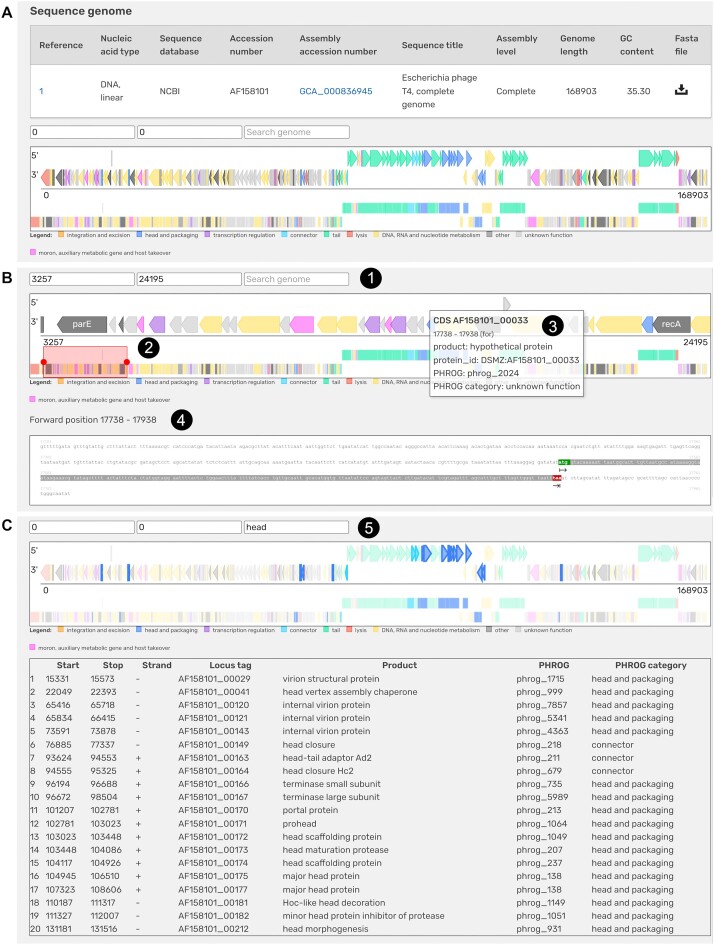
Screenshot of the genome viewer of *Escherichia* phage T4. (**A**) Primary view of the genome browser in the sequence genome section upon first access by the user. (**B**) Adapted browser view after zooming in a chosen genomic region (1) indicated by the red rectangular (2). Information on gene 33 is displayed in a mouse-over window (3), sequence information is provided separately below (4). (**C**) Results of a search for the term ‘head’ (5).

*Genome viewer*. A genome browser is implemented directly on the individual webpage of each strain if the genome sequence is available (Figure 2). In the current state Phage*Dive* contains 448 phage genomes that can be analysed with this browser tool. In order to standardize genome representation, the annotation was renewed using Prokka version 1.14.6 and the PHROG database (Prokaryotic Virus Remote Homologous Groups) (16), a constantly growing library of viral protein families generated by using a new clustering approach based on remote homology detection. Bacteriophage genomes generally harbour a lot of genes with unknown function. Using this library allows to add further annotations that are missed by standard annotation tools. The graphical display is based on the PHROG annotation, with genes individually coloured by PHROG category to give an initial overview of the general genome organization including gene clusters for structural genes, replication, lysis and others.

The primary view displays the organization of the entire virus genome (Figure 2A). To navigate through the annotated sequence different parameters are available. Zoom into the genome (Figure 2B) is possible via two alternatives. The first option is to enter the genomic coordinates of the area to be zoomed (Figure 2B, label 1). The second is to generate a zoom manually by framing the area of interest on the genomic sequence at the bottom (Figure 2B, label 2). Each gene has a ‘mouse-over’ window containing its main information, which appears when the mouse pointer is hovering over the gene in question (Figure 2B, label 3). Displayed are the gene name, position of the gene in the genome, function, its corresponding PHROG number and the PHROG assigned gene category. In addition, clicking on each gene also gives access to its nucleotide sequence that can be copied and pasted (Figure 2B, label 4). This tool also offers a function to search for specific genes by name or annotation category (Figure 2C, label 5). Results are displayed in a table containing the following information: start position, end position, DNA strand, locus tag and function (gene product), PHROG no. and category which can be copied and pasted. In addition, a list of these results can be seen on the genome overview table (Figure 2C).

**Figure 3.**
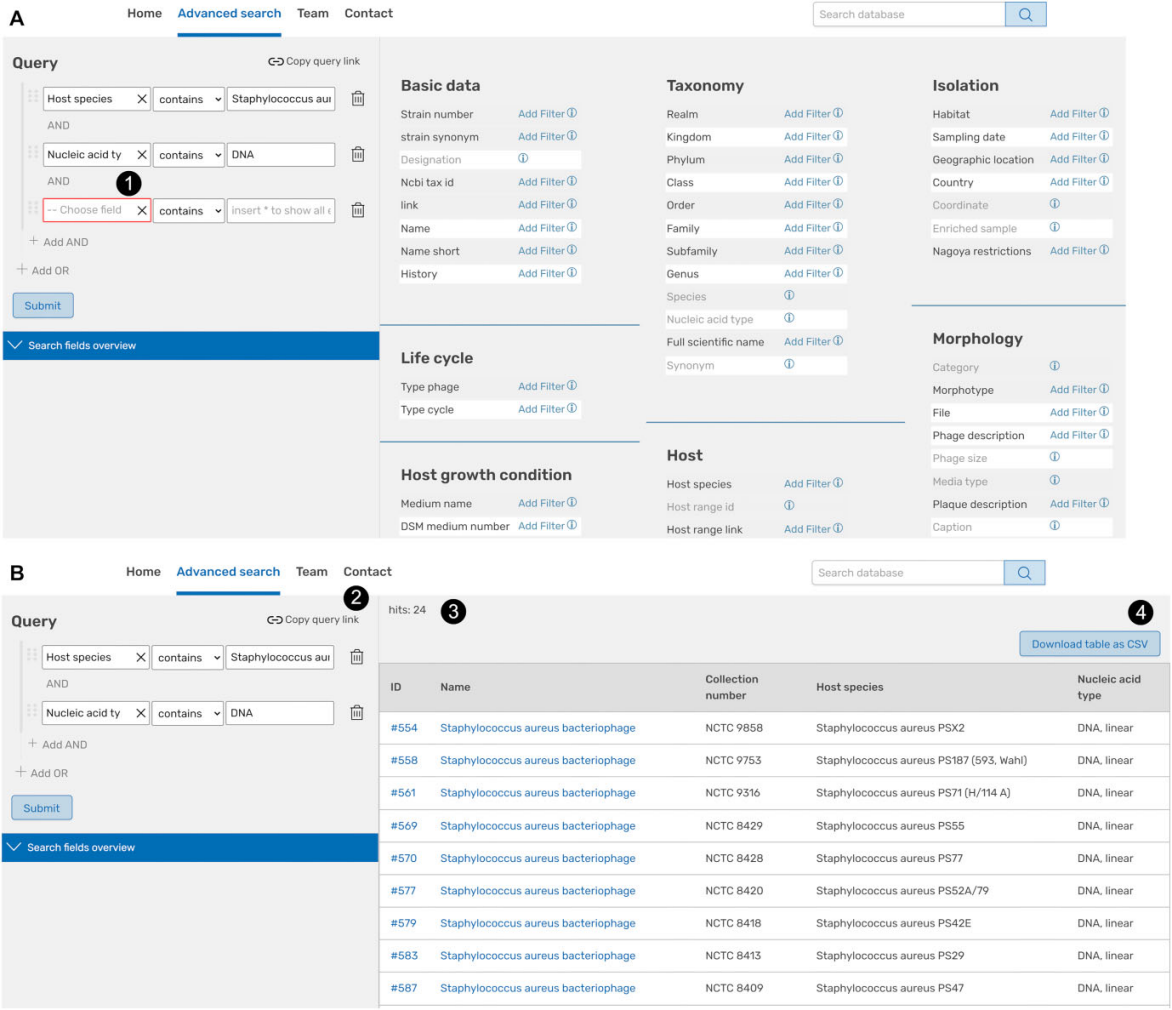
Advanced search tool. (**A**) Query specification with choice of data fields (1) used in Phage*Dive* to characterize viruses. (**B**) Result of a ‘Staphylococcus AND DNA’ search. A query link (2), the number of hits (3) and a download option (4) are provided for traceability.

*Advanced search*. The advanced search module is a key component for a refined search in Phage*Dive*. This tool is accessible through the menu on the top of each page. To retrieve the specific data of interest, the dataset can be accessed through queries that combine several data fields and analyse all entries for the virus strains. The query builder is located on the left-hand side of the tool page (Figure 3). Users are able to combine up to five data fields in an ‘AND’ combined query and even up to 15 data fields by connecting up to three groups using ‘AND’ or ‘OR’ operators (Figure 3, label 1). For each of these data fields to be combined a user can choose from a list of 72 options. Depending on the data field, input can be provided as text (e.g. plaque description), a numeric field (e.g. temperature) or from a selection field via a dropdown menu (e.g. biosafety level). For text fields, users are assisted by an autocomplete function providing suggestions of possible search terms. To start exploring data provided in the data fields, inserting the wildcard ‘*’ will allow users to retrieve all data available. As a result, a list of viruses corresponding to the request will be presented on the right (Figure 3B) with the number of hits noted above (Figure 3, label 3). The query can be bookmarked or shared using the ‘copy query link’ (Figure 3, label 2). The result list can be downloaded in a csv format table using the button on the upper right (Figure 3, label 4). In the same way the complete list of viruses included in Phage*Dive* is also accessible. The viruses are listed in alphabetical order and by culture collection (starting with the Félix d’Hérelle collection). The retrieved data set is restricted to the data fields queried.

## Discussion

Phage*Dive* is a new important resource in the domain of prokaryotic viruses designed to centralise all the data available for individual virus strain. Overall Phage*Dive* currently provides access to 11 331 data points for 1167 phage strains. It is based on a solid foundation of data originating from culture collections with the latter offering direct physical access to a wide variety of viruses and host strains. At the moment Phage*Dive* harbours viral data for members of at least 181 officially ICTV classified genera in 31 families (e.g. *Ackermannviridae* or *Herelleviridae*) in 6 classes over 4 realms (mostly *Duplodnaviria*) (Supplementary Figure S1). Additionally, the viruses in Phage*Dive* cover host organisms from 7 different phyla (14 classes, 35 orders, 103 genera). A major strength of Phage*Dive* is the breadth of the data space provided, notably the availability of plaque and/or TEM pictures, as well as multiple links to the various resources used (e.g. VHRdb for the host range, NCBI for genomes or ICTV for taxonomy). Though databases like e.g. PhageScope (9) might offer data from higher numbers of phages, this data is extracted (i) from sequences only and does not reflect any information from wet lab work like for instance propagation conditions (medium, temperature, need of bivalent cations) that might be valuable requirements for setting up experimental systems and (ii) the majority of analysed viruses is not available for practical experiments. Phage*Dive* provides direct, easy access to standardized data. The consequent standardization of data allows the use of tools to reliably search and analyse the information on phage strains and conduct highly precise searches to find phages with specific properties. For instance, during the SARS-CoV-2 pandemic, researchers looked for model organisms that shared features with the virus like nucleic acid material or size and came up with *Pseudomonas* phage phi6 (20) which might also have been the result for a Phage*Dive* search. Based on the information in Phage*Dive*, candidates for phage mixtures for different experimental approaches can be identified that e.g. share same host strains or originate from the same habitat, but can be differentiated by other categories like morphotype or genomic properties. The implementation of the advanced search tool and an extended interlinkage network with the various data providers make Phage*Dive* a highly robust source of information on prokaryotic viruses. With the integration of a genome viewer, genomes can be analysed more easily, providing a quick overview of their composition and organization. The integrated search function allows the fast and easy identification of specific genes of interest, e.g. endolysins or other enzymes, identifiable by standardized annotation. Various options for saving or downloading search results are provided. To take this a step further, an API will be implemented in the future to provide programmatic access to the continuous growing database. By applying the Creative Commons Attribution license (CC BY), an open access to the data is granted, ensuring the reuse of the data in the science community.

In the future, Phage*Dive* will be expanded in two directions. Firstly, the database will be enriched in parallel to the growth of the DSMZ phage collection and also through the integration of new datasets from external research groups and other phage banks. This will also include the targeted extension with phages for underrepresented organisms like anaerobic organisms or cyanobacteria. Secondly, new data fields will be added to complete the metadata sections, in particular by including transcriptomic and virus receptor data.

## Supplementary Material

gkae878_Supplemental_File

## Data Availability

All the data are publicly available at https://phagedive.dsmz.de licensed under CC BY 4.0. All data can be downloaded freely without restrictions, the only restriction being that the origin of the data must be properly cited when it is used in other works.

## References

[1] Adriaenssens E.M. , Roux S., Brister J.R., Karsch-Mizrachi I., Kuhn J.H., Varsani A., Yigang T., Reyes A., Lood C., Lefkowitz E.J. et al. Guidelines for public database submission of uncultivated virus genome sequences for taxonomic classification. Nat. Biotechnol. 2023; 41:898–902. 37430074 10.1038/s41587-023-01844-2 PMC10526704

[2] Ackermann H.-W. Bacteriophage taxonomy. Microbiol. Austr. 2011; 32:90–94.

[3] Dion M.B. , Oechslin F., Moineau S. Phage diversity, genomics and phylogeny. Nat. Rev. Micro. 2020; 18:125–138. 10.1038/s41579-019-0311-5 32015529

[4] Roux S. , Brum J.R., Dutilh B.E., Sunagawa S., Duhaime M.B., Loy A., Poulos B.T., Solonenko N., Lara E., Poulain J. et al. Ecogenomics and potential biogeochemical impacts of globally abundant ocean viruses. Nature. 2016; 537:689–693. 27654921 10.1038/nature19366

[5] Williamson K.E. , Fuhrmann J.J., Wommack K.E., Radosevich M. Viruses in soil ecosystems: an unknown quantity within an unexplored territory. Annu Rev Virol. 2017; 4:201–219. 28961409 10.1146/annurev-virology-101416-041639

[6] Bolduc B. , Shaughnessy D.P., Wolf Y.I., Koonin E.V., Roberto F.F., Young M. Identification of novel positive-strand RNA viruses by metagenomic analysis of archaea-dominated Yellowstone Hot Springs. J. Virol. 2012; 86:5562. 22379100 10.1128/JVI.07196-11 PMC3347303

[7] Breitbart M. , Hewson I., Felts B., Mahaffy J.M., Nulton J., Salamon P., Rohwer F. Metagenomic analyses of an uncultured viral community from human feces. J Bacteriology. 2003; 185:6220–6223. 10.1128/JB.185.20.6220-6223.2003 PMC225035 14526037

[8] Koonin E.V. , Krupovic M., Dolja V.V. The global virome: how much diversity and how many independent origins?. Environ. Microbiol. 2023; 25:40–44. 36097140 10.1111/1462-2920.16207

[9] Wang R.H. , Yang S., Liu Z., Zhang Y., Wang X., Xu Z., Wang J., Li S.C. PhageScope: a well-annotated bacteriophage database with automatic analyses and visualizations. Nucleic Acids Res. 2024; 52:D756–D761. 37904614 10.1093/nar/gkad979 PMC10767790

[10] Gao N.L. , Zhang C., Zhang Z., Hu S., Lercher M.J., Zhao X.M., Bork P., Liu Z., Chen W.H. MVP: a microbe-phage interaction database. Nucleic Acids Res. 2018; 46:D700–D707. 29177508 10.1093/nar/gkx1124 PMC5753265

[11] Lamy-Besnier Q. , Brancotte B., Ménager H., Debarbieux L. Viral Host Range database, an online tool for recording, analyzing and disseminating virus-host interactions. Bioinformatics. 2021; 37:2798–2801. 33594411 10.1093/bioinformatics/btab070 PMC8428608

[12] Russell D.A. , Hatfull G.F. PhagesDB: the actinobacteriophage database. Bioinformatics. 2017; 33:784–786. 28365761 10.1093/bioinformatics/btw711 PMC5860397

[13] Turner D. , Shkoporov A.N., Lood C., Millard A.D., Dutilh B.E., Alfenas-Zerbini P., van Zyl L.J., Aziz R.K., Oksanen H.M., Poranen M.M. et al. Abolishment of morphology-based taxa and change to binomial species names: 2022 taxonomy update of the ICTV bacterial viruses subcommittee. Arch. Virol. 2023; 168:74. 36683075 10.1007/s00705-022-05694-2 PMC9868039

[14] Reimer L.C. , Sardà Carbasse J., Koblitz J., Ebeling C., Podstawka A., Overmann J. BacDive in 2022: the knowledge base for standardized bacterial and archaeal data. Nucleic Acids Res. 2022; 50:D741–D746. 34718743 10.1093/nar/gkab961 PMC8728306

[15] Koblitz J. , Halama P., Spring S., Thiel V., Baschien C., Hahnke R.L., Pester M., Overmann J., Reimer L.C. MediaDive: the expert-curated cultivation media database. Nucleic Acids Res. 2023; 51:D1531–D1538. 36134710 10.1093/nar/gkac803 PMC9825534

[16] Seemann T. Prokka: rapid prokaryotic genome annotation. Bioinformatics. 2014; 30:2068–2069. 24642063 10.1093/bioinformatics/btu153

[17] Terzian P. , Olo Ndela E., Galiez C., Lossouarn J., Pérez Bucio R.E., Mom R., Toussaint A., Petit M.A., Enault F. PHROG: families of prokaryotic virus proteins clustered using remote homology. NAR Genom. Bioinform. 2021; 3:lqab067. 34377978 10.1093/nargab/lqab067 PMC8341000

[18] Nishimura Y. , Yoshida T., Kuronishi M., Uehara H., Ogata H., Goto S. ViPTree: the viral proteomic tree server. Bioinformatics. 2017; 33:2379–2380. 28379287 10.1093/bioinformatics/btx157

[19] Hulo C. , de Castro E., Masson P., Bougueleret L., Bairoch A., Xenarios I., Le Mercier P ViralZone: a knowledge resource to understand virus diversity. Nucleic Acids Res. 2011; 39(Database issue):D576–D582. 20947564 10.1093/nar/gkq901 PMC3013774

[20] Gomes M. , Bartolomeu M., Vieira C., Gomes A.T.P.C., Faustino M.A.F., Neves M.G.P.M.S., Almeida A. Photoinactivation of Phage Phi6 as a SARS-CoV-2 model in wastewater: evidence of efficacy and safety. Microorganisms. 2022; 10:659. 35336234 10.3390/microorganisms10030659 PMC8954818

