# Supplementary material for: PhageDive: the comprehensive strain database of prokaryotic viral diversity
Source: Nucleic Acids Res. 2024 Oct 7;53(D1):D819–25. doi: 10.1093/nar/gkae878 (PMC11701545; doi:10.1093/nar/gkae878)
Supplement: gkae878_Supplemental_File [file gkae878_supplemental_file.pdf]

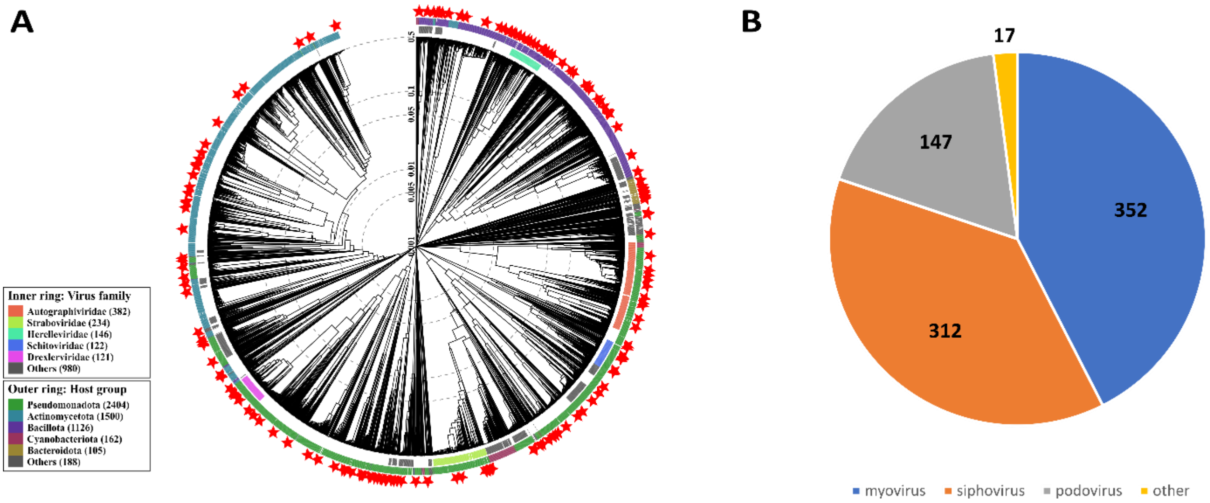

Figure S1. Overview of the phage diversity covered in Phage*Dive* in terms of phage and host taxonomy (A) and morphology (B). (A) Single members of officially classified ICTV genera included in Phage*Dive* are marked by stars using ViPTree. Viral families are coloured in the inner ring, host phyla are coloured on the outer ring. (B) Distribution of morphotypes among the phages in Phage*Dive* with available information on morphology.
